# Root microbiota alters response to root rot in *Rhododendron delavayi Franch*

**DOI:** 10.3389/fmicb.2023.1236110

**Published:** 2023-08-25

**Authors:** Jing Tang, Yufeng Xiao, Xiaorong Xu, Ming Tang, Ximin Zhang, Yin Yi

**Affiliations:** ^1^Key Laboratory of State Forestry Administration on Biodiversity Conservation in Karst Mountainous Areas of Southwestern China, School of Life Sciences, Guizhou Normal University, Guiyang, China; ^2^State Key Laboratory of Plant Physiology and Development in Guizhou Province, Guizhou Normal University, Guiyang, China

**Keywords:** root-microbiota, root rot, *Rhododendron delavayi Franch*, niche, diseased plant

## Abstract

Root microbiota have a significant effect on plant health. However, the role of root microbiota in the resistance of *Rhododendron* against root rot is not known. In this study, we employed amplicon 16S and ITS sequencing to investigate the bacterial and fungal communities associated with four distinct niches (bulk soil, rhizosphere, rhizoplane, and endosphere) of both healthy and diseased *Rhododendron* plants in the Baili Rhododendron nature reserve in China. The amplicon data analysis identified 182 bacterial genera and 141 fungal genera that were impacted by root rot across all niches. Specifically, the rhizoplane appeared to exert a selective gating effect, resulting in a reduction in the complexity of bacterial communities, but not fungal communities, in wild *Rhododendron delavayi Franch* roots. Nevertheless, the stress induced by root rot led to alterations in the root microbiota and compromised the gating function of the rhizoplane, thereby significantly increasing the complexity of the bacterial community within the plant root. In the root tissue following root rot outbreak, the relative abundance of the pathogenic species *Pezicula brunnea* and *Diaporthe helianthi* was enriched by as much as 6.13% and 1.71%, respectively. These findings provide novel insights into the contribution of enrichment of root-associated microbiota to wild plant hosts under the disease stress of root rot. The root rot-causing pathogenic fungi may interact with beneficial bacteria and induce plants to send out “cry for help” signals, which may encourage the specific assembly of microbiota. In the *Rhododendron delavayi Franch* root microbiota, we found 23 potentially beneficial microbes. Notably, certain beneficial bacteria, such as *Sporolactobacillus* and *Stenotrophomonas*, were found to accumulate in the rhizoplane and endosphere under root rot disease stress. Overall, our results lend support to our hypothesis that *Rhododendron* recruits protective microbes as a strategy to suppress root rot outbreaks. Future endeavors in isolating beneficial microbes capable of mitigating root rot have the potential to enhance plant resilience against root diseases.

## 1. Introduction

Plants and soil are intertwined with each other, with microbes playing a crucial role in this interconnection. Plant microbiota have a significant effect on plant health, both on the root (below ground) and the phyllosphere (above ground) (Berlec, 2012). The below-ground niche encompasses the bulk soil, rhizosphere, rhizoplane, and endosphere. Bulk soil is the soil in which the plant roots live, except for the rhizosphere’s soil. The rhizoplane is the root surface, which is in contact with the rhizosphere soil (Berendsen et al., 2012). The endosphere consists of microbes that live inside plant tissues for at least part of their life cycle (Zgadzaj et al., 2016). The microbial communities of the rhizoplane and endosphere are affected by the plant root. The microbes of the rhizosphere, rhizoplane, and endosphere make up the root microbiota (Zhang et al., 2017). The soil microbial community is thought to be responsible for biological processes that are necessary for maintaining healthy soil and suppressing plant diseases (Trivedi et al., 2020; Liu et al., 2021). The root microbiotas play a significant role in many aspects of plant growth and health, including supplying plants with nutrition (Vandenkoornhuyse et al., 2015), stimulating seed germination (Bulgarelli et al., 2013), promoting abiotic stress resistance (Lebeis, 2014), eliciting systemic plant defenses (Bulgarelli et al., 2015), improving antibiosis functions against pathogens (Berg et al., 2016). In addition, root microbes promote plant growth by acting as a source and sink for nutrients (Puglisi et al., 2012). The microbiome is an integral part of the plant-host combination, which is a “holobiont” (Vandenkoornhuyse et al., 2015). In addition, studies have shown that plant species are closely related to root microbiota, and different plant species have host-specific root microbiota (Berendsen et al., 2012; Oh et al., 2012). Given its importance, the assembly mechanism of the root-associated microbiome is important for plant growth and health.

A growing body of research has shown that the root microbiome helps plants to resist stresses, such as drought, nutrition, and disease (Egamberdieva et al., 2017; Larousse et al., 2017; Santos-Medellín et al., 2017; Staley et al., 2017). Several recent studies have revealed that the rhizosphere microbiome provides a defense against severe disease outbreaks (Gao et al., 2021; Zhou et al., 2022), such as the soil-borne disease caused by *Fusarium* (Mendes et al., 2018; Zhu et al., 2023) and *Rhizoctonia* (Chapelle et al., 2016). In addition, these studies mainly used annual plants including Arabidopsis, soybean, cucumber, wheat, tomato, sugar beet, barley, and rice (Zhang et al., 2017; Cregger et al., 2018; Carrión et al., 2019; Fernández-González et al., 2020), as well as a few perennial plants, including citrus and populous (Cregger et al., 2018). Compared with annual plants, there are fewer studies on the assembly of microbial communities to resist stresses in perennial plants. In fact, perennial plants undergo a long interaction cycle with their root microbiome, including complicated root growth patterns and long and variable environmental factors (Zhang et al., 2017). Collectively, it is generally agreed that there is a tight linkage between the plant root microbial communities and plant disease, and plants recruit disease-suppressive microbes to respond to pathogen attacks. However, there are few reports about root microbiota after an outbreak of root rot. In particular, root rot in *Rhododendron* has never been reported. Moreover, the effects of different plants on the composition of root microbiota are highly complex and dynamic (Yin et al., 2021). Hence, it remains unclear how plants shape the change of root microbiota to respond to the root rot disease.

*Rhododendron delavayi Franch* is one species of the genus *Rhododendron*, the flowers of which are beautiful and brightly colored. The species is a highly attractive perennial ornamental tree. The genus *Rhododendron* is the largest genus in the family Ericaceae, containing approximately 1,000 species, all of which are evergreen woody shrubs or trees (Ma et al., 2016). Baili Rhododendron nature reserve is one of the largest Rhododendron Primeval Forests, in which the flower belt of original rhododendrons extends for 125.8 km^2^. Unfortunately, it is worrying that there is a continuous area of outbreak of *Rhododendron delavayi Franch* root rot, and this even caused the death of *rhododendron*. Since plants are affected by a variety of biotic and abiotic factors to assemble their root microbiota, we hypothesized that *Rhododendron* would recruit protective microbes to suppress root rot outbreaks. We also expected that the “recruit “effect is related to the below-ground niche in which the root microbes are located. Further, there is a lack of research on the changes and assembly of fungal communities in roots under disease stress. And considering that fungal communities are more responsive to plant change (Dassen et al., 2017), we emphasize the necessity of exploring alterations within fungal communities. To elucidate some of these inquiries, we employed amplicon sequencing targeting the V4 region of the 16S rRNA gene and the ITS1 region to investigate the microbial composition within four distinct niches: bulk soil, rhizosphere, rhizoplane, and endosphere. Our study focused on both healthy and diseased *Rhododendron delavayi Franch* specimens cultivated in natural soil. Within this context, we provide a comprehensive account of the shifts occurring in the root-associated microbiome of *Rhododendron delavayi Franch* during root rot infection, while also inferring the underlying mechanisms by which root microorganisms influence plant root rot.

## 2. Materials and methods

### 2.1. Sample collection

Rootstock was collected from 36 normal (N) and 36 diseased (D) *Rhododendron delavayi Franch* grown at the Baili Rhododendron nature reserve in China (located at 27°08′38 N, 105°45′45E) (Supplementary Figure S1). Six randomly selected *Rhododendron delavayi Franch* rootstock were mixed into one sample. All of the diseased *Rhododendron delavayi Franch* roots were rotten. All of the normal root samples were collected from wild, natural *Rhododendron delavayi Franch* trees without root rot symptoms. We analyzed the root microbiota (the rhizosphere, rhizoplane, and endosphere) and the microbe of bulk soil in the mixed soil of *Rhododendron delavayi Franch* root side (Supplementary Figure S2). Bulk soil is composed of a mixture of the remainder soil, except for ~3 cm topsoil. The rhizosphere compartment was composed of less than 5 mm of soil tightly adhering to the root surface, and we gently swept and collected the soil using a brush. The root surface was disinfected by repeated wiping three times with 75% alcohol. We carefully divided the rhizoplane from the endosphere using a scalpel. All of the samples were collected in May 2016.

### 2.2. DNA extraction and sequencing

We vortexed 5 g roots and/or attached soils in 5 mL phosphate-buffered saline (PBS) solution (pH 7.2). The microbiota from the bulk soil, rhizosphere, rhizoplane, and endosphere were removed by high-speed vortex in the PBS buffer. The roots, soil, and supernatant were discarded, leaving only the resulting slurry. Using approximately 100 μL of the resulting slurry, DNA was extracted by MOBIO PowerSoil^®^ DNA Isolation Kit (12888-50, United States). To minimize DNA extraction bias, a sample was extracted three times and mixed. We dissolved the extracted metagenome in 60 μL TE buffer, then quantified the DNA ND1000 and stored it at −80°C. The V4 region of the 16S rRNA gene and the ITS1 region of the fungal internal transcribed spacer were amplified using PCR and sequenced using the Illumina HiSeq platform (da C. Jesus et al., 2010; Lopez-Velasco et al., 2011). The PCR primer information is in the additional file (Supplementary Table S1).

### 2.3. Sequence analysis

The sequence analysis was carried out by QIIME (Version 1.7.0) (Caporaso et al., 2010). Paired-end reads were merged using FLASH (Magoč and Salzberg, 2011), and quality filtering was performed (Bokulich et al., 2013). The chimera sequences were removed using the UCHIME algorithm (Edgar et al., 2011). Using Uparse software (Edgar et al., 2011), sequences with ≥97% similarity were assigned to the same OTUs. Species were annotated with the GreenGene Database^1^ (DeSantis et al., 2006) and UNITE database^2^ (Kõljalg et al., 2013). OTUs abundance information was normalized using a standard of sequence number corresponding to the sample with the least sequences. Analysis of alpha diversity and beta diversity was performed based on normalized OTU abundance information (Ju and Zhang, 2015).

### 2.4. Statistical analyses

Alpha diversity is calculated by analyzing the complexity of species diversity for a sample through Shannon and Simpson. We used the Kruskal-Wallis H test to analyze differences in alpha diversity between the different groups. Beta diversity [Bray-Curtis distances, weighted UniFrac (WUF) metric] analysis was used to evaluate differences in samples in terms of species complexity. To elucidate the difference in the microbial communities between the different groups, the constrained principal coordinate analysis (CPCoA) and principal coordinate analysis (PCoA) of Bray-Curtis distances at the OTU levels were calculated (Zgadzaj et al., 2016). Additionally, using PERMANOVA (Adonis), we measured the effect significances on β-diversity. All these indices were calculated by the Vegan R package (Oksanen et al., 2011) and visualized by the ggplot2 R package. In order to investigate significant differences in microbial abundance, we conducted a likelihood ratio test (LRT) using the DESeq2 R packages (Love et al., 2014; McMurdie and Holmes, 2014). The Venn diagrams were plotted with the VennDiagram R package. We analyzed root microbiota networks from normal and diseased sampling. Using the Pearson correlation coefficient in the WCGNA R package, the network was based on the 37 pathogenic and beneficial microbes.

## 3. Results

### 3.1. Comprehensive information on microbial communities

We sequenced the microbial community of the *Rhododendron delavayi Franch* rootstock at four niches, rhizosphere, rhizoplane, endosphere, and bulk soil. In total, we generated 8,263,696 high-quality paired-end clean reads of the V4 region of the 16S rRNA gene and 7,522,048 reads of the ITS1 gene. The average reads were 86,080 and 78,355 for each per sample, which was not significantly different in the samples. After merging and filtering, the minimum reads of bacteria (fungi) were 158 (218), and the maximum was 390 (278). We prepared rarefaction curves, which showed that the increased number of OTUs reached a plateau (Supplementary Figure S3), with good coverage above 99.3%. Therefore, our regeneration of microbial communities for all samples was satisfactory, and there were no sequencing differences. For the microbiota in the below-ground parts of wild-grown *Rhododendron delavayi Franch*, there are 41 phyla and 338 genera of bacteria and 6 phyla and 230 genera of fungus. Of these, 9 bacterial phyla and 3 fungal phyla made up more than 1% of the sample. The most abundant bacterial phyla were *Proteobacteria* (55.70% ± 19.56), *Acidobacteria* (21.26% ± 12.31), and *Actinobacteria* (9.61% ± 1.81). Meanwhile, the most abundant fungal phyla were *Zygomycota* (38.57% ± 7.95) and *Basidiomycota* (33.51% ± 8.01).

### 3.2. Distinct and overlapping microbial communities in four spatially separable root niches

**Figure 1:**
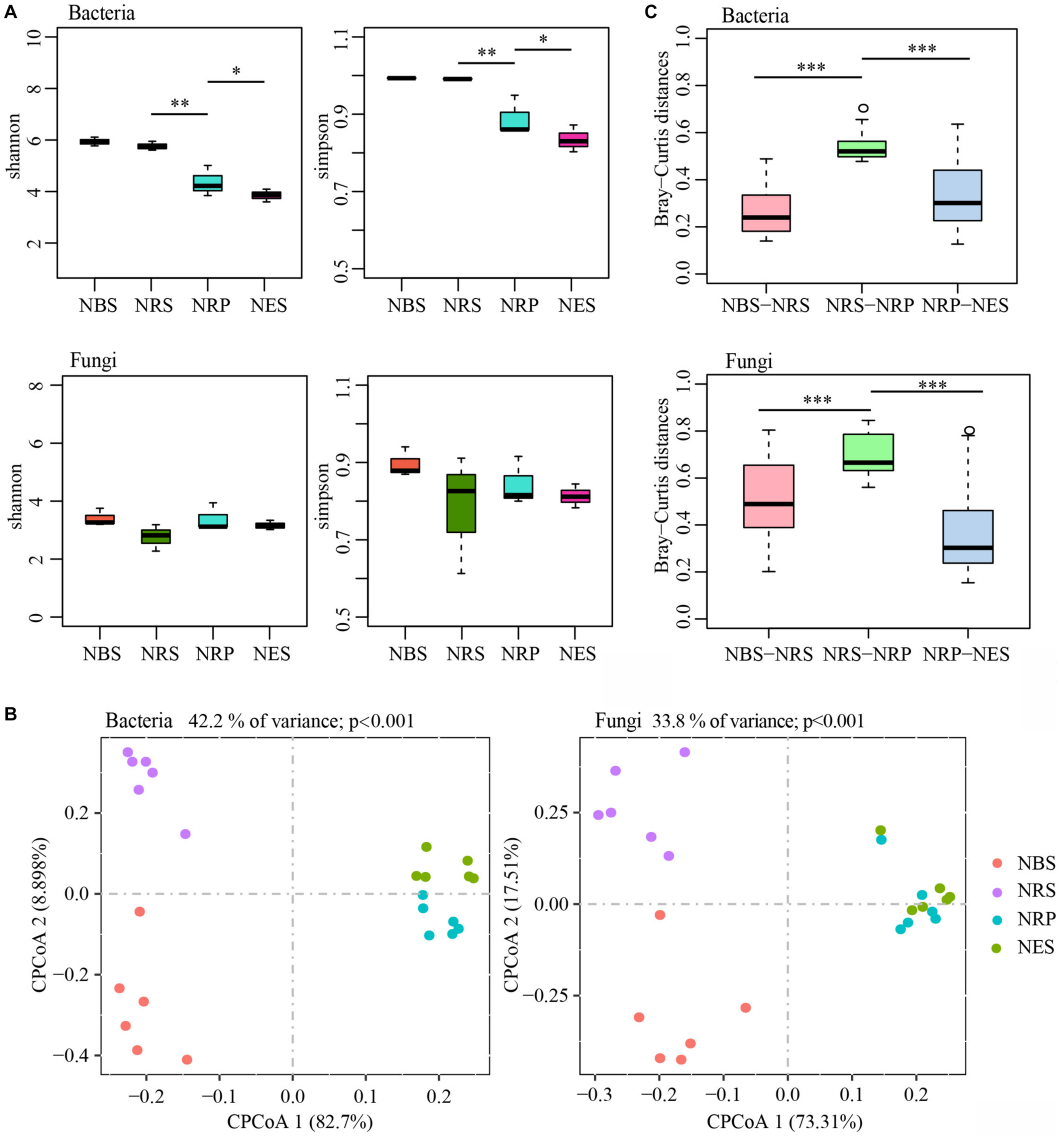
The diversity of bulk soil and root microbiota are separable by niches. **(A)** Difference analysis of alpha diversity estimated of the microbial communities (*p < 0.05; **p < 0.01). **(B)** The constrained principal coordinate analysis (CPCoA) plot of Bray–Curtis distances between samples constrained by niches (Each point corresponds to a different sample colored by niches.). **(C)** Difference analysis of Bray–Curtis distances between two adjacent niches of samples (***p < 0.001; Tested by Kruskal-Wallis H test.). N: normal wild *Rhododendron delavayi Franch*; BS: Bulk soil; RS: Rhizosphere; RP: Rhizoplane; ES: Endosphere.

We analyzed the bacterial and fungal microbiota from four separate niches of wild *Rhododendron delavayi Franch*: the bulk soil, rhizosphere, rhizoplane, and endosphere. Comparisons of α-diversity revealed a diversity variation of bacterial communities, but not of fungal communities, from the bulk soil to the endosphere (Figure 1A). Compared with the rhizoplane and endosphere, the mean α-diversity of bacterial communities was significantly higher in the rhizosphere and bulk soil. In addition, the lowest α-diversity of endosphere bacterial communities was significantly lower than that of rhizoplane. However, the α-diversity of bacterial communities between the rhizosphere and bulk soil was not significantly different. Interestingly, the difference in α-diversity in fungal communities between the four niches was not statistically significant. These results indicated the amount and uniformity of bacterial species largely decreased from rhizosphere to endosphere.

To assess the effect of niches on the assembly of microbiota, we compared the Bray-Curtis distances and performed a constrained principal coordinate analysis (CPCoA) (Figure 1B). This analysis revealed a clear differentiation of samples belonging to the bulk soil, rhizosphere, rhizoplane, and endosphere that explains as much as 42.2% of the overall variance of the bacterial data (*p* < 0.001) and 33.8% of the fungal data (*p* < 0.001). Furthermore, the rhizoplane and endosphere microbial communities were remarkably divergent from the rhizosphere and bulk soil (separation by first component). We also found that the Bray-Curtis distances between rhizosphere and rhizoplane were the highest (Figure 1C). In addition, PERMANOVA (Adonis) using the WUF distance supports the CPCoA results that the microbial communities vary significantly between niches (bacteria: explained 41.56%, *p* < 0.01; fungi: explained 25.64%, *p* < 0.05). These results indicated that the bacterial and fungal microbiota associated with the bulk soil, rhizosphere, rhizoplane, and endosphere of wild *Rhododendron delavayi Franch* roots vary, and the largest difference was between the rhizosphere and rhizoplane.

Transformations of the root-associated colonizing microbiota across four niches once again provide evidence that root niches play a selective role in microbiota assembly (Ofek-Lalzar et al., 2014; Edwards et al., 2015). Moreover, the rhizoplane may play a selective gating role that largely reduces the complexity of bacterial communities but not fungal communities.

### 3.3. The microbial taxa of differential abundance in root niches

In order to identify niche-associated microbial communities, we evaluated differentially abundant taxa between two adjacent niches using a likelihood ratio test. At the OTU, phylum, and genus levels, the analysis was performed to assess the extent of these changes at different taxonomic ranks.

*OTU-level analysis.* According to ≥97% sequence identity, we clustered the high-quality clean reads into 2,452 reliable bacterial OTUs and 996 fungal OTUs (low-abundance OTUs (≤5 total counts) were discarded). We found that 106 bacterial OTUs and 37 fungal OTUs presented in bulk soil, 9 bacterial OTUs and 19 fungal OTUs in the rhizosphere, 93 bacterial OTUs and 33 fungal OTUs in the rhizoplane, and 25 bacterial OTUs and 15 fungal OTUs in the endosphere (Supplementary Figure S4A). Across all differential abundance contrasts, we detected 1,053 differentially abundant bacterial OTUs and 426 fungal OTUs between niches (Supplementary Figure S4B and Supplementary Table S2). Overall, in bacterial communities, the most differentially abundant OTUs were the enriched OTUs in the rhizoplane (accounting for 42.26%), followed by the depleted OTUs in the rhizoplane (25.45%). Interestingly, in fungal communities, the most differentially abundant OTUs were the depleted OTUs in the rhizoplane (30.99%), followed by the enriched OTUs in rhizoplane (23.94%). There were approximately 7% bacterial and fungal differentially abundant OTUs enriched in the rhizoplane and depleted in the endosphere. The rhizosphere and rhizoplane presented the highest number of differentially abundant OTUs in bacteria, followed by bulk soil and rhizosphere. Accordingly, in fungi, the greatest difference was between the rhizosphere and rhizoplane, followed by between rhizoplane and endosphere (Supplementary Figure S4C). The relative abundance of differential OTUs between rhizosphere and rhizoplane was more than 60% (Supplementary Figure S4D). These results also indicated that the root microbiota is associated with niches. Most root microbiota changed between the rhizosphere and the rhizoplane. In addition, the rhizoplane may play a selective gating role that induces a change of over 60% in the root microbiota.

**Figure 2:**
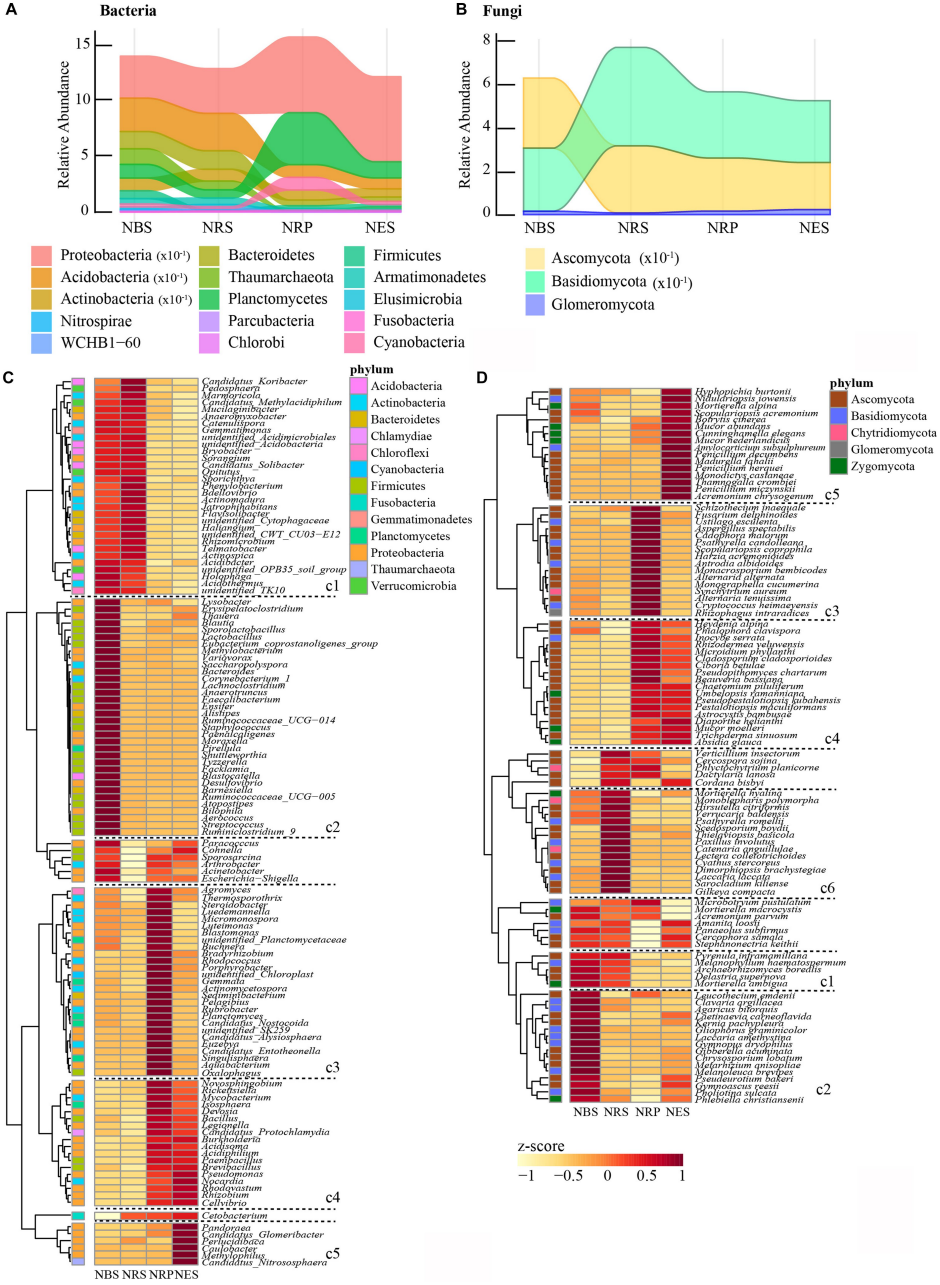
Bulk soil and root-associated microbial communities are separable by niches in phyla and genus level. Relative abundance of bacterial **(A)** and fungal **(B)** phyla significantly changed with niches. Heatmap showing the abundance of bacterial **(C)** and fungal **(D)** genus associated with niches. (The *z*-scores represented the abundance of genus. cl cluster indicated the genus enriched bulk soil and rhizosphere; c2: enriched bulk soil; c3: enriched rhizoplane; c4: enriched rhizoplane and endosphere; c5: enriched endosphere; c6: enriched rhizosphere. The significant difference of microbial abundance between niches tested by a likelihood ratio test. *p* < 0.05). N: normal wild *Rhododendron delavayi Franch*; BS: Bulk soil; RS: Rhizosphere; RP: Rhizoplane; ES: Endosphere.

*Phylum and genus-level analysis*. Overall, there were 32 bacterial and 5 fungal phyla in the four niches of *Rhododendron delavayi Franch* root. There were significantly differential abundances of 15 bacterial and 3 fungal phyla (Figures 2A,B). In bacterial communities, the rhizoplane and endosphere had a greater relative abundance of *Proteobacteria* than the rhizosphere or bulk soil. Moreover, the endosphere had more *Planctomycetes* and *Cyanobacteria*. In contrast, except for *Firmicutes* and *Fusobacteria*, the remaining phyla were of higher relative abundance in the rhizosphere and bulk soil. The relative abundance of *Firmicutes* was significantly lower in the rhizosphere, rhizoplane, and endosphere; *Fusobacteria* was notably enriched in these niches. In fungal communities, the rhizosphere had a greater relative abundance of *Basidiomycota* than other niches. In contrast, the rhizosphere had the lowest relative abundance of *Glomeromycota*. In addition, the relative abundance of *Ascomycota* was notably lower in the rhizoplane and endosphere. At the genus level, there were 123 bacterial and 98 fungal genera associated with differential niches (Figures 2C,D). Most genera (60, c2) were significantly enriched in the bulk soil; in contrast, the fewest genera (22, c5) were significantly enriched in the endosphere. Moreover, most of the genera enriched in the bulk soil were classified as *Firmicutes*. In the endosphere, we observed a significant enrichment of *Pandoraea*, *Perlucidibaca*, *Caulobacter*, *Methylophilus*, *Hyphopichia burtonii*, *Nidulariopsis iowensis*, *Mortierella alpina*, *Scopulariopsis acremonium*, *Botrytis cinerea*, *Mucor abundans*, *Cunninghamella elegans*, *Mucor nederlandicus*, *Amylocorticium subsulphureum*, *Penicillium decumbens*, *Madurella fahalii*, *Penicillium herquei*, *Monodictys castaneae*, *Thamnogalla crombiei*, *Penicillium miczynskii,* and *Acremonium chrysogenum* as compared to other niches. Thirty-six genera (c1) were significantly enriched in bulk soil and rhizosphere soil, and accordingly, 36 genera (c4) were significantly enriched in the rhizoplane and endosphere. The differential genera within Acidobacteria were persistently enriched in bulk soil and rhizosphere. Forty-three genera (c3) were significantly enriched in rhizoplane. Their result also indicated a coherence of niches across taxonomic ranks.

### 3.4. Root microbiota exhibit disease-mediated compositional shifts caused by root rot

**Figure 3:**
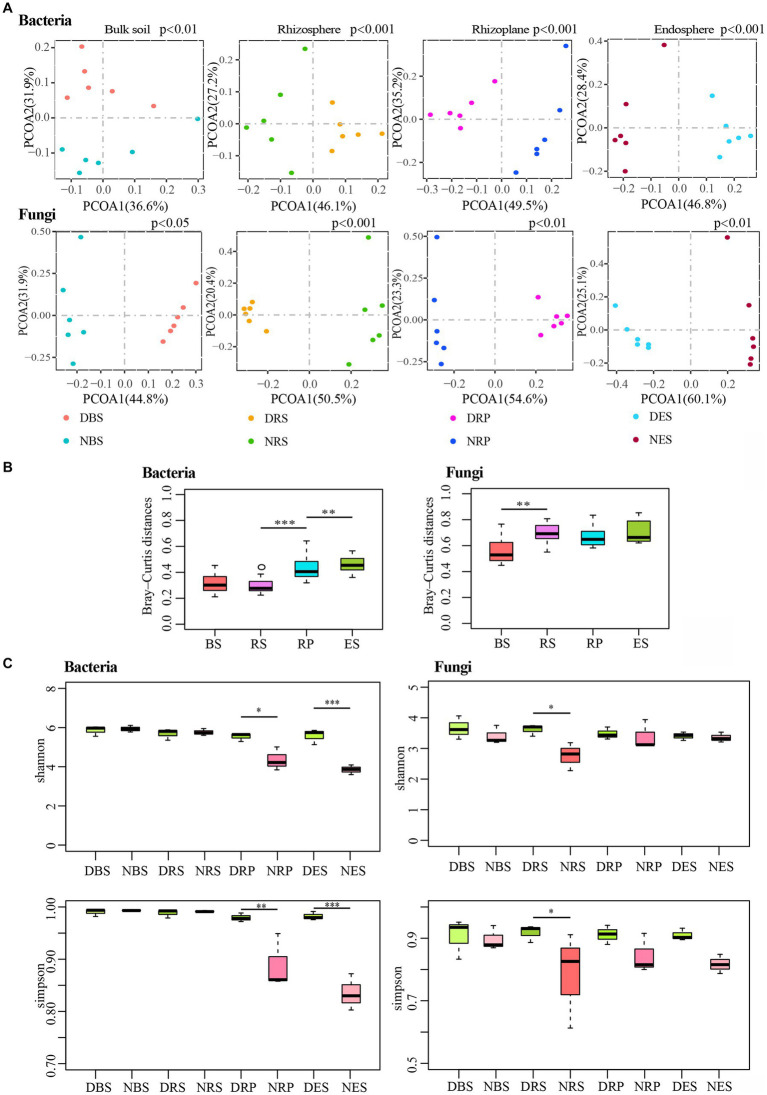
Root rot disease-mediated changed the diversity of bulk soil and root microbial communities. **(A)** Unconstrained principal coordinate analyses (PCoA) using the Bray–Curtis distances indicates that the bulk soil and root microbiota separated by root rot disease. (*p*: Tested by PERMANOVA). **(B)** Difference analysis the differential distance of diseased and normal root-microbiota between four niches using Bray–Curtis distances. (**p < 0.01, ***p < 0.001. Tested by Kruskal-Wallis H test.). **(C)** Compared the alpha diversity between the diseased and normal group by Kruskal-Wallis H test. (*p < 0.05; **p < 0.01; ***p < 0.001.). BS: Bulk soil; RS: Rhizosphere; RP: Rhizoplane; ES: Endosphere. N: normal samples; D: diseased samples.

In order to examine the root microbiota response to root rot disease, we sampled the soil and roots of diseased and normal plants (Supplementary Figure S2). We analyzed the impact of root rot disease on the four niches by performing principal-coordinate analysis (PCoA) on Bray-Curtis distances (Figure 3A). The first principal coordinate (PCOA1) displayed the communities separated across disease-normal in each niche, except for the bacterial community of bulk soil, in which it was the second principal coordinate. In addition, a PERMANOVA analysis of weighted UniFrac distances also indicated that the microbial communities of the four niches significantly varied between the diseased and normal groups (Supplementary Table S3). To compare the differential distances of diseased and normal root-microbiota between the four niches, we used the Kruskal-Wallis H test to analyze the difference in Bray-Curtis distances (Figure 3B). The distance of bacterial communities between diseased and normal endosphere was furthest, and the distance of rhizoplane was notably further than bulk soil and rhizosphere. Correspondingly, the distance of fungal communities between diseased and normal rhizosphere was further than in bulk soil. These results showed that root rot disease significantly impacted the *Rhododendron delavayi Franch* root bacterial and fungal communities in all four of the niches. Meanwhile, the effect of root rot disease on root microbiota was greater than that of bulk soil. By comparing the α-diversity between the diseased and normal group, the bacterial α-diversity of rhizoplane and endosphere in the diseased group were higher than those of the normal group; at the same time, the fungal α-diversity of the rhizosphere of the diseased group was higher than that of normal (Figure 3C). α-diversity measurements also indicated that root rot disease significantly impacts the complexity of root microbiota. We conclude that the stress caused by root rot disease resulted in a change of root microbiota and reduced gating function of the rhizoplane, which significantly increased the complexity of bacterial communities in the root of the plant.

In addition, the differentially abundant OTUs caused by disease stress are distributed in all of the niches (Supplementary Figure S5A and Supplementary Table S4). The numbers of differentially abundant OTUs accounted for almost one-third of total OTUs in the four niches (Supplementary Figure S5B). The CPCoA of the Bray-Curtis distances indicated a clear differentiation of diseased samples belonging to the bulk soil, rhizosphere, rhizoplane, and endosphere (Supplementary Figure S6A). Moreover, in the diseased plant sample, the rhizoplane and endosphere microbial communities were also remarkably divergent from the rhizosphere and bulk soil (Supplementary Figure S6B). Furthermore, analyzing the cluster of microbial communities on four niches in normal and diseased samples, root microbiota exhibit disease-mediated compositional shifts (Supplementary Figure S6C). In addition, these changes still not entirely broke the spatially separable niches. While wild-grown *Rhododendron delavayi Franch* disease was caused by root rot disease, a clear differentiation of root microbiota from four niches still existed.

### 3.5. Interactions between spatially separable niches and disease caused by root rot disease

**Figure 4:**
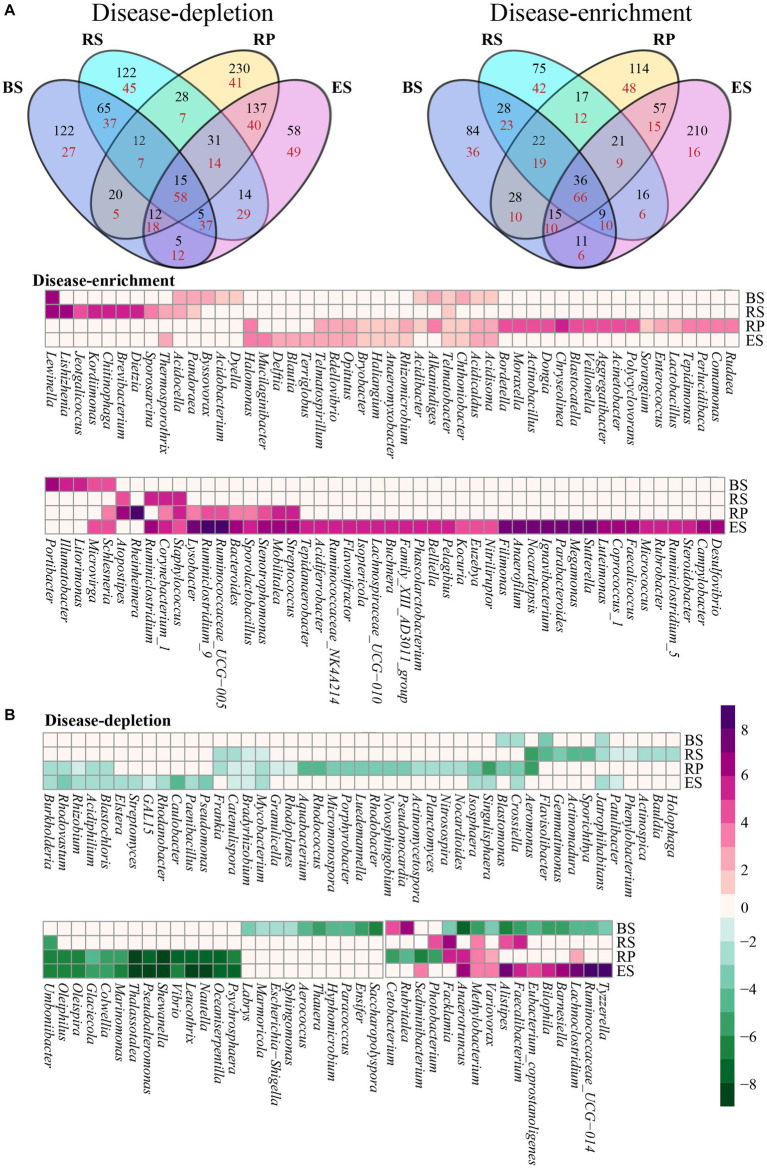

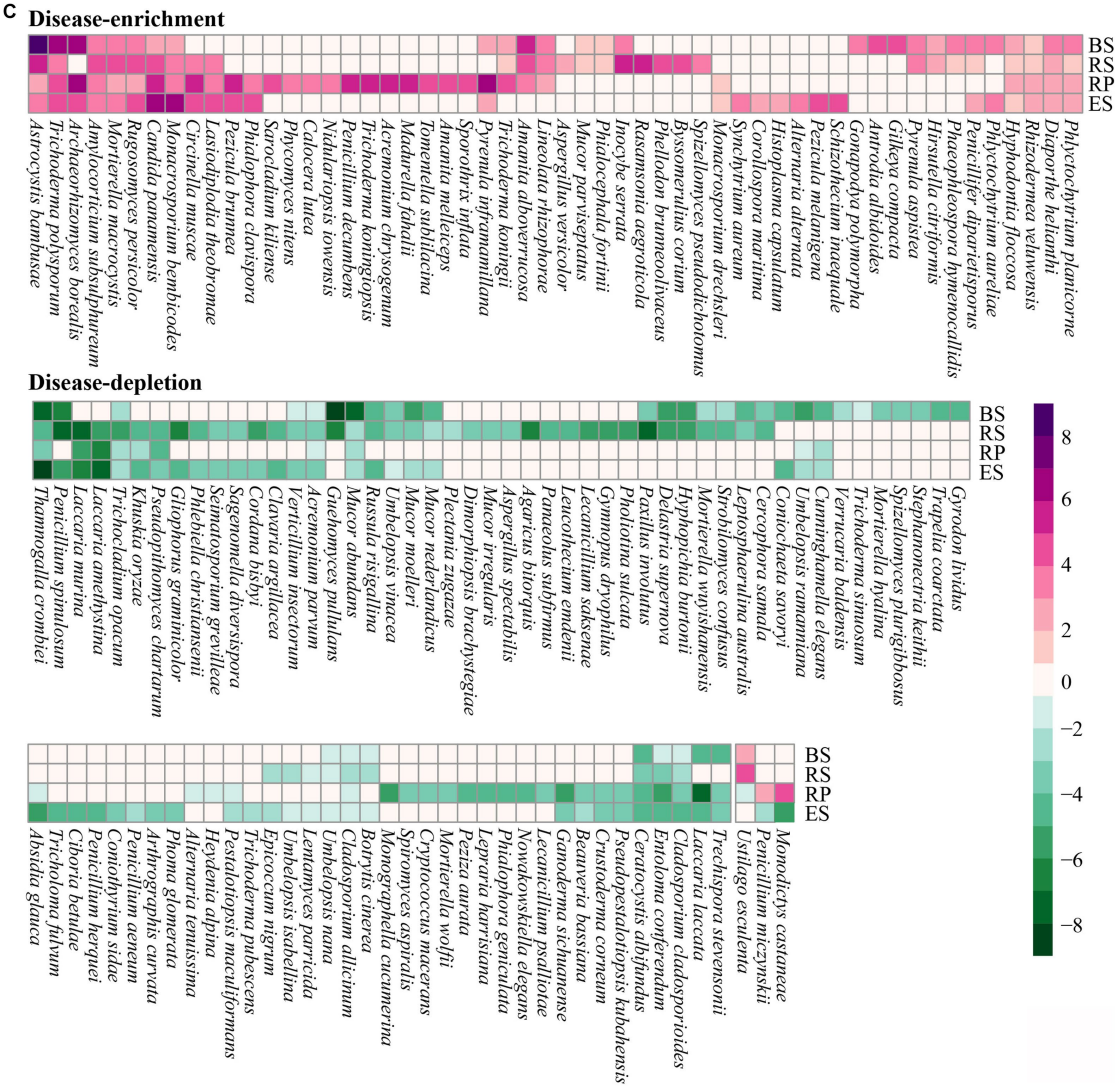
Four niches microbial communities exhibited a distinct disease-response for root rot. **(A)** A Venn diagram comparing differentially disease-depleted and disease-enriched microbial OTUs in four niches. Disease-responsive bacterial **(B)** and fungi **(C)** taxa (*p* < 0.05) in each niches. The color of the cell indicated the log2 fold change in relative abundance between diseased and normal groups. An increase tend purple, while a decrease tend toward green. The plot displays a genus detected as significantly affected by root rot disease in at least one niches. BS: Bulk soil; RS: Rhizosphere; RP: Rhizoplane; ES: Endosphere.

We analyzed the interaction between disease and niches by exploring the overlap between the disease-responsive OTUs detected in the four niches (Figure 4A). Overall, the OTUs affected by disease overlapped across all of the niches. However, in bacterial communities, the majority of disease-responsive OTUs specifically resided in a single niche (depletion 60.73%, enrichment 65.01%). In contrast, in fungal communities, the overlap of all niches had the most OTUs affected by the disease (depletion 13.62%, enrichment 20.12%). While there was overlap between the disease-responsive OTUs in all of the niches, more nonspecifically distributed fungal OTUs were depleted or enriched in all of the niches compare with bacterial OTUs. In addition, we found interactions between niches and disease at the genus level. In bacterial communities, 338 genera were annotated in the four niches, of which 182 genera affected by disease overlapped across all niches (Figure 4B). Nearly half of these genera were solely distributed in one niche (60 disease-enriched genera, 39 disease-depleted genera). Among them, most disease-enriched genera were especially concentrated in the endosphere (30 genera), such as *Campylobacter*, *Desulfovibrio*, *Steroidobacter,* and so on. This was followed by the rhizoplane (17 genera), such as *Comamonas*, *Rudaea*, *Perlucidibaca,* and so on. Correspondingly, most disease-depleted genera were distributed in the rhizoplane (15 genera), such as *Granulicella*, *Rhodoplanes*, *Aquabacterium*. Interestingly, most of the differential genera that varied in the four niches presented consistency (except for 16 genera). Just 12 genera were depleted in the niche of bulk soil and enriched in another niche, such as *Facklamia*, *Anaerotruncus*, *Methylobacterium*, *Variovorax*, *Alistipes*, *Faecalibacterium*, *Bilophila*, *Barnesiella*, *Eubacterium_coprostanoligenes*, *Lachnoclostridium*, *Cetobacterium*, *Photobacterium*, *Rubritalea*, *Sediminibacterium*, *Ruminococcaceae_UCG-014*. and *Tyzzerell* depleted in the rhizoplane and enriched in another niche. In fungal communities, 141 out of 230 genera that overlapped across all of the niches were affected by disease (Figure 4C). Nearly a third of the genera were distributed in only one niche (25 disease-enriched genera, 33 disease-depleted genera), which was less than for bacterial communities. Similarly, almost all differential fungal genera changed in the four niches also presented consistently (except for *Ustilago esculenta*, *Penicillium miczynskii*, and *Monodictys castaneae*). These results also indicated that some genera are depleted or enriched in particular compartments during root rot disease, instead of some genera affected by disease migrating from one niche to other niches.

## 4. Discussion

Root microbiota is well-known to connect with plant health, and a plant being invaded by pathogens can promote the enrichment of a group of beneficial root microbes (Berendsen et al., 2018). In this study, we demonstrated how disease caused by root rot drives microbiome composition and diversity alterations in all of the root niches. More than a third of root microbiota that are associated with the plant were altered with root rot, which overlapped across all niches. However, compared with the fungal community, more bacterial taxa were depleted or enriched in any given niche. These results are consistent with the reconstruction of root microbial communities under drought stress (Santos-Medellín et al., 2017). It is further supported that, under the stress of extreme environments or disease, the root microbiota may be altered by plant calls. Moreover, we speculate that the altered root microbiota might contribute to plant survival (Bakker et al., 2018). Therefore, future studies are needed to focus on the function of the discrepant microbial community. At the function level, root microbiota can be pathogenic, beneficial (Mendes et al., 2013), or neutral (Kaestli et al., 2012; Bai et al., 2022). Pathogens trigger plant diseases and, as such, are used for the diagnosis of plant diseases (Alvarez, 2004). In addition, some beneficial microbes in the plant microbiota benefit the plant, such as rhizobia, one function of which is benefitting resisting plant disease. In the *Rhododendron delavayi Franch* root microbiota, we found 23 potentially beneficial microbes by document retrieval (Supplementary Table S5). These beneficial microbes are selectively concentrated in distinct niches of normal roots. In the rhizoplane and endosphere, a large number of beneficial microbes was enriched, including *Acidiphilium*, *Bacillus*, *Burkholderia*, *Paenibacillus*, *Brevibacillus*, *Pseudomonas*, and *Rhizobium* (Figure 2). Among these, the relative abundance of *Burkholderia* and *Pseudomonas* accounted for 28.08% and 24.58%. The functions of *Burkholderia* include nitrogen fixing (Alvarez, 2004), increasing phosphorus solubilization and efficiency (Alvarez, 2004), producing pyrrolnitrin (Raaijmakers et al., 2002), and inhibiting the growth of fungal pathogens such as *Fusarium* (Raaijmakers et al., 2002). Interestingly, we separated a large number of pathogenic *Fusarium* sp. from the petals of *Rhododendron delavayi Franch* blossom rot (another ongoing and unpublished study). In fact, in the Baili Rhododendron nature reserve, the blossom rotten disease always infects the normal *Rhododendron delavayi Franch* petals. Similarly, the function of *Pseudomonas* also includes increasing access to nutrients for plants (Duarah et al., 2011), promoting plant growth (seedling length, root, and shoot length, fresh and dry weight) (Vyas et al., 2010), increasing the activity of peroxidase and phenylalanine ammonia-lyase in plant tissues (Sharma et al., 2007), and suppressing plant disease (such as foliar anthracnose) (Tripathi et al., 2006). In the Baili Rhododendron nature reserve, the foliar anthracnose of *Rhododendron delavayi Franch* is also a common disease, just like blossom rotten disease. These suggest that petal and leaf infection with pathogens purposefully signals to the root to promote the growth of beneficial microbial species in the root microbiota. We considered that, in the natural *Rhododendron delavayi Franch*, the assemblage of root microbiota (especially *Burkholderia* and *Pseudomonas*) is promoted by floral and foliar diseases. The periodic disease pressure of plant leaves and petals is one of the important reasons for microbial assembly in perennial tree roots.

Almost all of the predicted beneficial microbes, except for the *Brevibacillus*, *Bacillus*, *Microbacterium,* and *Sphingomonas*, changed significantly due to the disease pressure of root rot (Figure 4). Interestingly, we found that some of the beneficial bacteria associated with the disease stress of root rot accumulated in the root system after root rot, and others were depleted. The beneficial bacteria *Acidocella*, *Delftia*, *Acinetobacter*, *Opitutus*, *Methylobacterium*, *Micrococcus*, *Sporolactobacillus*, *Staphylococcus,* and *Stenotrophomonas* were enriched. Conversely, *Acidiphilium*, *Bradyrhizobium*, *Brevibacillus*, *Burkholderia*, *Ensifer*, *Frankia*, *Micromonospora*, *Paenibacillus*, *Pseudomonas*, *Rhizobium*, and *Streptomyces* were obviously depleted. We speculated that these may be caused by two reasons: (1) when plant root rot occurred, plant roots recruited beneficial root microbiota; (2) there exist antagonistic interactions between pathogenic and beneficial root microbiota.

**Figure 5:**
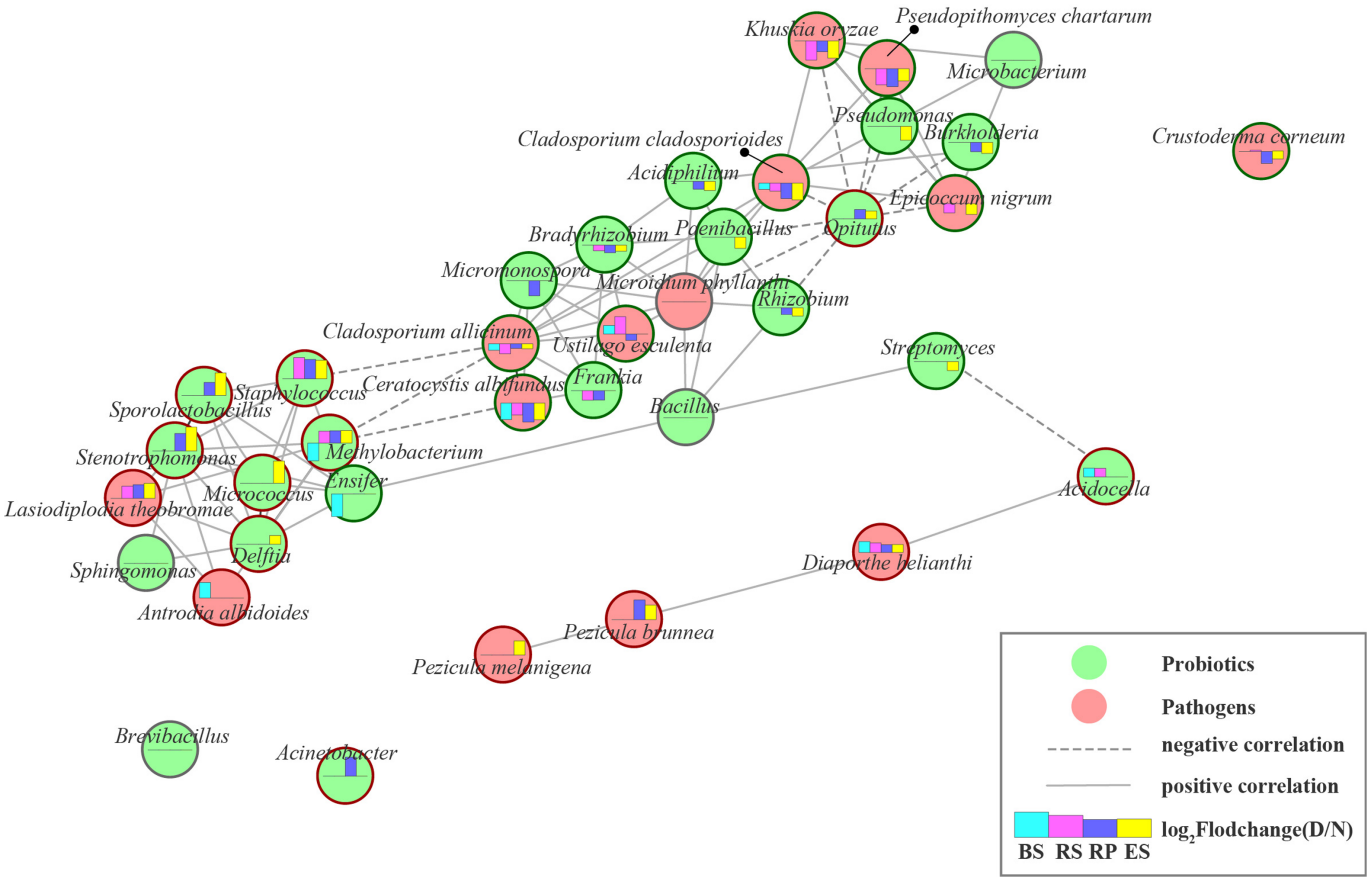
The beneficial and pathogenic microbial interaction networks. Nodes represent individual beneficial or pathogenic microbe; edges represent significant Pearson correlation coefficient (*r* > 0.6, *p* < 0.001) between two microbes from normal to diseased plant in rhizocompartment. Edges length represent the magnitude of correlation coefficients (1-|*r*|). The longer the line, the smaller the correlation coefficients. Green circular ring indicate that the microbe strongly decreased under plant disease-stress, corresponding, red circular ring indicate increase. Moreover, gray circular ring indicated unchanged. Histograms displays the log2 fold change of genus relative abundances between diseased and normal groups. Column upward indicated the microbe significantly enriched in the diseased group. BS: Bulk soil; RS: Rhizosphere; RP: Rhizoplane; ES: Endosphere. N: normal samples; D: diseased samples.

In the diseased *Rhododendron delavayi Franch* root-microbiota, we found 14 potentially pathogenic fungi by document retrieval (Supplementary Table S6). From normal to diseased plants, the beneficial and pathogenic microbes established a complex and close interaction network between the whole root system (Figure 5), except for *Brevibacillus*, *Acinetobacter*, *Crustoderma corneum*. After the disease of *Rhododendron delavayi Franch* caused by root rot, the abundance of 7 pathogenic fungi significantly decreased, including *Ceratocystis albifundus*, *Cladosporium allicinum*, *Cladosporium cladosporioides*, *Crustoderma corneum*, *Epicoccum nigrum*, *Khuskia oryzae*, and *Pseudopithomyces chartarum*. In the interaction network, they were positively correlated with beneficial microbes such as *Micromonospora*, *Frankia*, *Bradyrhizobium*, *Acidiphilium*, *Paenibacillus*, *Rhizobium*, *Pseudomonas*, *Burkholderia,* and *Microbacterium*. In contrast, *Opitutus* was strongly negatively related to them. Remarkably, the pathogenic fungi *Diaporthe helianthi*, *Lasiodiplodia theobromae*, *Pezicula brunnea*, and *Pezicula melanigena* were notably enriched in the diseased root system. In particular, *Pezicula brunnea* and *Diaporthe helianthi* proliferated, resulting in their relative abundance as high as 6.13% and 1.71% in the diseased root tissue. We infer that *Pezicula brunnea* and *Diaporthe helianthi* may be pivotal pathogens in *Rhododendron delavayi Franch* disease caused by root rot. Unfortunately, neither of the two pathogens has been reported in the *Rhododendron delavayi Franch*. In the interaction network, there was a positive relationship between them and *Acidocella*. Moreover, *Streptomyces* and *Acidocella* showed a significant competitive inhibition relationship. *Streptomyces* prevents root and sprout diseases (Berg, 2009) and protects against soil pathogens such as *Pythium*, *Fusarium*, and *Phomopsis* (Maksimov et al., 2011). In fact, recent experiments have shown that there is an antagonistic relationship between plant-beneficial bacteria, and the coexisting interactions between these plant-beneficial bacteria were clarified (Molina-Santiago et al., 2019).

**Figure 6:**
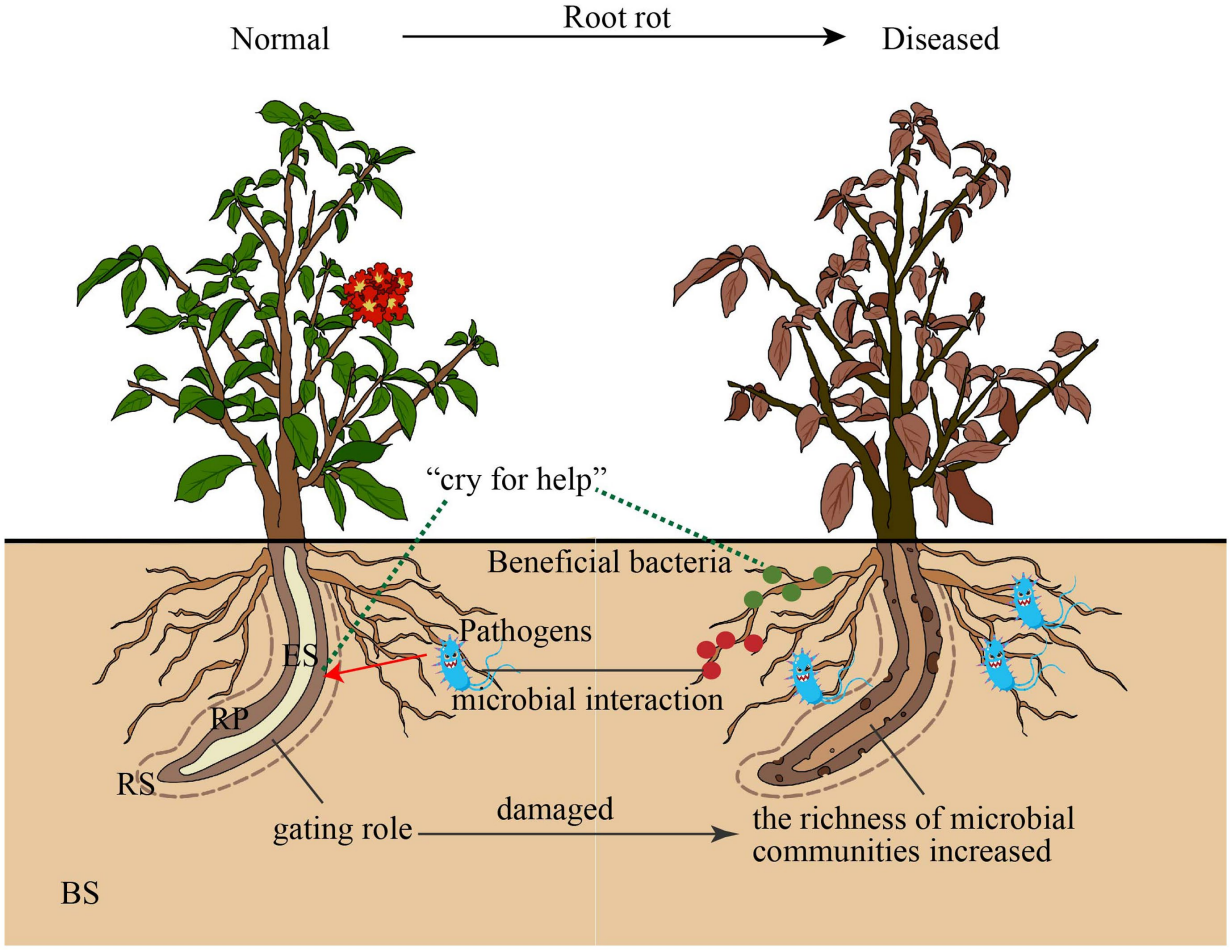
Proposed model for bulk soil and root microbiota communities responded root rot disease. BS: Bulk soil; RS: Rhizosphere; RP: Rhinoplane; ES: Endosphere.

This study provides a detailed characterization of the effect of root rot disease stress on the microbial community of four niches in wild *Rhododendron delavayi Franch* root. Here, we describe a mechanistic model through which bulk soil and root microbiota respond to plant disease caused by root rot. Our combined results support the mechanistic model (Figure 6): (1) the rhizoplane exerts a selective gating effect, which can be damaged by root rot. In the below-ground portion of field-grown *Rhododendron delavayi Franch*, differential niches of roots selectively recruit specific microbial communities (Figure 1). In addition, the rhizoplane plays a potential and critical gating role that controls the microbial community in the root (Zhang et al., 2017), which significantly reduces the richness of bacterial communities in rhizoplane and endosphere. However, the decrease in the fungal communities is not significant (Figure 1). Root diseases lead to a significant shift in root microbiota composition (Larousse et al., 2017). After *Rhododendron delavayi Franch* root rot disease, bulk soil, rhizosphere, rhizoplane, and endosphere microbial communities are all significantly altered (Figure 3). We infer that the gating function of the rhizoplane may be damaged in the process of plant illness caused by root rot. The richness of bacterial communities in the rhizoplane and endosphere helps to confirm these hypotheses. In addition, we speculate that (2) once root rot breaks out, root signaling generated by the plant produces a “cry for help” that encourages the specific assembly of particular microbiota in four niches. Some recent studies have shown that plants can “cry for help” from their root microbiota: upon foliar pathogen attack, plants are selectively enriched for beneficial microbes and microbial activities (Raaijmakers et al., 2002; Bakker et al., 2018). In the natural *Rhododendron delavayi Franch*, the assemblage of root microbiota (especially beneficial bacteria *Burkholderia* and *Pseudomonas*) is also promoted by the floral and foliar diseases (Figure 2). Furthermore, in the diseased *Rhododendron delavayi Franch* root, the microbial communities at four niches are still different (Supplementary Figure S6). Much of the microbiota is depleted or enriched in particular compartments during root rot disease. Our results suggest that root infection with a pathogen is also beginning to deliver the “help signal.” Finally, (3) from normal to diseased plants, pathogens in the root microbiota affect a potential interaction in the microbiota/beneficial bacteria community. We have established the complex and close interaction network between the pathogenic and beneficial microbes in the root-microbiota (Figure 5). There is a direct or indirect co-occurrence or competition between almost all pathogens and beneficial bacteria.

Nevertheless, it is crucial to acknowledge that the mechanistic model proposed in this study requires additional validation to adequately address several key inquiries. Firstly, it is necessary to isolate the specific pathogenic and beneficial microbes involved. Subsequent experiments should aim to elucidate the precise mechanisms of interaction between these microbes during disease development stages. Lastly, in order to enhance our understanding of the impact of the plant’s “help signal” on the root microbiota, further investigations are needed to confirm the effects of root exudation compounds on the suppression of root rot.

## 5. Conclusion

In our investigation, we have observed that the presence of root rot disease imparts a transformative effect on the composition and diversity of the root microbiota in *Rhododendron delavayi Franch*. Notably, the rhizoplane appears to assume a selective gating role, leading to a marked reduction in the complexity of bacterial communities, although fungal communities remain largely unaffected. Importantly, disease-induced stress disrupts the gating function of the rhizoplane, resulting in a significant increase in the complexity of bacterial communities within the plant’s root system. Additionally, a plausible explanation for the response of the root microbiota to disease lies in the collaborative influence of the plant’s “help signal” and the potential interactions between pathogenic and beneficial microbes. However, the examination of this hypothesis warrants further investigation, requiring the isolation of both pathogens and beneficial bacteria to ascertain its validity.

## Data availability statement

The datasets presented in this study can be found in online repositories. The names of the repository/repositories and accession number(s) can be found below: https://www.ncbi.nlm.nih.gov/sra, PRJNA1008699.

## Author contributions

JT: writing and methodology. YX: data analysis. XX: samples collected. MT: functional annotations. XZ: visualization. YY: conceived and designed the experiment. All authors contributed to the article and approved the submitted version.

## Funding

This research was funded by the National Natural Science Foundation of China-Guizhou Provincial People’s Government Karst Science Research Center Project (U1812401), Science and Technology Support Project of Guizhou Province (QKHZC[2021]YB459), the Natural Science Foundation of China (NSFC) (32260393); Key Laboratory of Environment-Friendly Management on Alpine Rhododendron Diseases and Pests of Institutions of Higher Learning in Guizhou Province ([2022]044) and Karst Mountain Ecological Security Engineering Research Center, grant number [2021]007.

## Conflict of interest

The authors declare that the research was conducted in the absence of any commercial or financial relationships that could be construed as a potential conflict of interest.

## Publisher’s note

All claims expressed in this article are solely those of the authors and do not necessarily represent those of their affiliated organizations, or those of the publisher, the editors and the reviewers. Any product that may be evaluated in this article, or claim that may be made by its manufacturer, is not guaranteed or endorsed by the publisher.

## Supplementary material

The Supplementary material for this article can be found online at: https://www.frontiersin.org/articles/10.3389/fmicb.2023.1236110/full#supplementary-material

## Footnotes

^1^ http://greengenes.lbl.gov/cgi-bin/nph-index.cgi

^2^ https://unite.ut.ee/
